# Neural stem cells deriving from chick embryonic hindbrain recapitulate hindbrain development in culture

**DOI:** 10.1038/s41598-018-32203-w

**Published:** 2018-09-17

**Authors:** Yuval Peretz, Ayelet Kohl, Natalia Slutsky, Marko Komlos, Stas Varshavsky, Dalit Sela-Donenfeld

**Affiliations:** Koret School of Veterinary Medicine, The Robert H. Smith Faculty of Agriculture, Food and Environment, The Hebrew University of Jerusalem, Rehovot, Israel

## Abstract

Neural stem cells (NSCs) are self-renewing multipotent cells that line the neural-tube and generate all the nervous system. Understanding NSC biology is fundamental for neurodevelopmental research and therapy. Many studies emphasized the need to culture NSCs, which are typically purified from mammalian embryonic/adult brains. These sources are somewhat limited in terms of quantity, availability and animal ethical guidelines. Therefore, new sources are needed. The chick is a powerful system for experimental embryology which contributed enormously to neurodevelopmental concepts. Its accessibility, genetic/molecular manipulations, and homology to other vertebrates, makes it valuable for developmental biology research. Recently, we identified a population of NSCs in the chick hindbrain. It resides in rhombomere-boundaries, expresses Sox2 and generates progenitors and neurons. Here, we investigated whether these cells can recapitulate hindbrain development in culture. By developing approaches to propagate and image cells, manipulate their growth-conditions and separate them into subpopulations, we demonstrate the ordered formation of multipotent and self-renewing neurospheres that maintain regional identity and display differential stem/differentiation/proliferation properties. Live imaging revealed new cellular dynamics in the culture. Collectively, these NSC cultures reproduce major aspects of hindbrain development *in-vitro*, proposing the chick as a model for culturing hindbrain-NSCs that can be directly applied to other neural-tube domains and species.

## Introduction

The central nervous system (CNS) is composed of billions of cells that vary in type, number and density across different domains^1,2^. The CNS originates from common progenitors located at the ventricular layer of the developing neural tube, known as neural stem cells (NSCs)^3^. NSCs have an ability to self-renew for many generations or to differentiate into an amplitude of neurons or supporting cells in response to external and internal cues^4^. As such, they serve as a constant source for new cells that develop within different brain and spinal cord regions in the embryo. In the adult, however, the generation of new neurons is extremely limited. Yet, many studies have confirmed the existence of few NSC reservoirs in the dentate gyrus of the hippocampus, subventricular zone (SVZ) in the walls of the lateral ventricles, as well as in other regions, that can undergo neurogenesis throughout life upon specific conditions^5^.

The proper balance between self-renewal and differentiation of NSCs is fundamental. Due to their huge potential for studying and treating CNS pathologies, strategies were developed to grow, trace and manipulate them in well-controlled *in vitro* systems^6^. Along the years, the conditions for culturing NSCs, maintaining them as multipotent progenitors or differentiating them into numerous derivatives improved significantly^7^. Remarkably, regardless of their origin, cultured NSCs typically form distinct free-floating compact entities termed neurospheres that have an ability to self-renew upon their dissociation into single cells. In addition, they consist of multipotent cells, which mimic the *in vivo* differentiation hierarchy; quiescent/slow proliferating NSCs are usually located in the sphere’s core, and mitotically-active progenitors undergo final differentiation into neurons or glia lineages upon migration towards its outer layers^8^. Neurospheres also tend to establish their unique extracellular-matrix microenvironment, which helps in maintaining their stemness^9^.

Along with many similar properties of neurospheres from different CNS origins, they do retain regional identity^10–12^. For instance, the SVZ contains large numbers of NSC that continually generate new neurons destined for the olfactory bulb (OB). Yet, isolation of NSCs from distinct regions along the SVZ will produce different types of OB neurons *in vitro*; NSCs from the dorsal SVZ will give rise to superficial granule cells and tyrosine hydroxylase expressing cells, whereas neurospheres originating from the ventral SVZ will produce deep granular cells and calbindin-positive neurons^13^. Similarly, hindbrain cells isolated from mice embryo (E12) or human embryos (E35–E60) were found to contain NSCs that give rise to stereotypic neurospheres in culture. These neurospheres preserved hindbrain-specific markers such as particular Hox genes and the neurotransmitter 5-hydroxytryptamine that is exclusive to hindbrain serotonergic neurons^14,15^.

In the young embryo, the hindbrain is transiently subdivided along its anterior-posterior axis into segments termed rhombomeres. Each rhombomere is a cell-lineage-restricted compartment which displays a unique pattern of gene expression, neural crest migration and neuronal differentiation^16,17^. This unique topography makes the hindbrain a landmark to investigate regional specification, patter formation and neurogenesis, as the template for the future brainstem. In the adult, the brainstem serves as a key relay hub that links the bilateral half sides of lower and upper CNS centers via an extensive network of neural pathways^15–19^. It controls vital functions such as breathing and heart pressure, as well as processes and transmits sensory inputs to regulate hearing, balance, proprioception, motor coordination and more. These neural assemblies are critically shaped by the rhombomere’s segmentation, that gives rise to both common and distinct neurons along their AP and dorsal-ventral axes^20–24^.

Recently, we identified new sites of NSCs in the chick embryonic hindbrain in specialized domains in-between rhombomeres, termed hindbrain boundaries. These repetitive zones contain quiescent/slow proliferating cells that express typical NSC markers such as Sox2, nestin and GFAP, which contribute amplifying progenitors and differentiating neurons to adjacent domains^25^. These cells are also able to form neurospheres *in vitro*^25^. In the current work, we further investigated whether hindbrain-derived neurospheres can recapitulate hindbrain development in culture. We explored the different conditions for neurosphere formation, maintenance and differentiation, and demonstrated their ability to self-renew, aggregate, divide, connect, and differentiate into distinct lineages, as well as to retain their regional identity. Using live imaging, we documented the highly dynamic nature of cells within and in-between neurospheres. We also unraveled different stem or differentiation properties of subpopulations of cells that were isolated and purified *in vitro*. Based on the huge homology of the embryonic hindbrain across species, together with our finding that hindbrain-derived NSCs reproduce major aspects of hindbrain development *in vitro*, this study presents the chick hindbrain as a powerful model for isolating and studying NSCs, that can be directly applied into other neural-tube domains and species.

## Results

### Formation of hindbrain-derived neurospheres is dependent upon growth medium and cell density

NSCs deriving from embryonic forebrain and spinal cord were well documented to generate multipotent self-renewing neurospheres in culture^26–31^. Yet, little data exists regarding the hindbrain. We have recently demonstrated the ability of cells originating from E3.5 (st.18 HH) chick hindbrains to give rise to spheres that express progenitor and differentiation markers^25^. Here we aimed to investigate the growth conditions and cellular dynamics through which hindbrain NSCs generate neurospheres that can differentiate into multiple lineages, to determine how well the hindbrain regional identity is maintained *in vitro*, and to identify whether purified hindbrain cell subpopulations display unique differentiation features in the culture.

**Figure 1:**
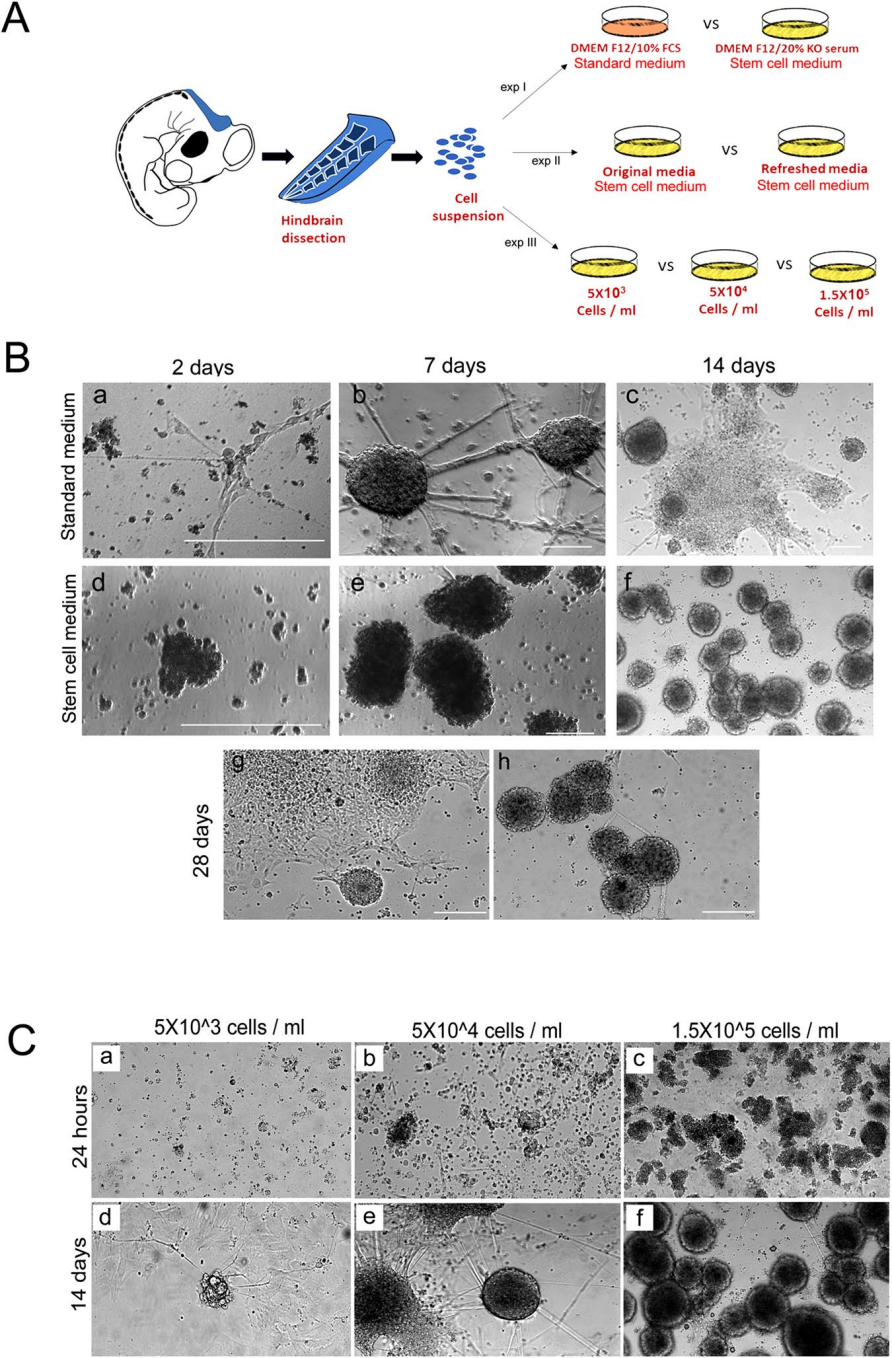
Formation of hindbrain spheres is dependent on growth media and cell density. (**A**) Scheme of experimental design showing culturing of cells from st.18 HH chick hindbrains using different protocols. (**B**) (a–f). Bright field views of cells cultured in standard (a–c) or stem cell (d–f) medium replenished every 3 days. Cultures were documented for up to 14 days. (g,h) Bright field views of cells cultured for 28 days in original stem cell medium or (g) upon medium replenishment every 3 days (h). (**C**) (a–f). Bright field views of cells cultured in increasing densities (5 × 10^3^–1.5 × 10^5^ cells/ml). Cells were documented after 24 hrs (a–c) and 14 days (d–f) in culture. Each image is a representative of 10 different cultures from three biological repeats. Each biological repeat included dissection of 35–40 embryonic hindbrains. Scale bars in Ba,d = 75 um. In all other images scale bar = 50 um.

**Figure 2:**
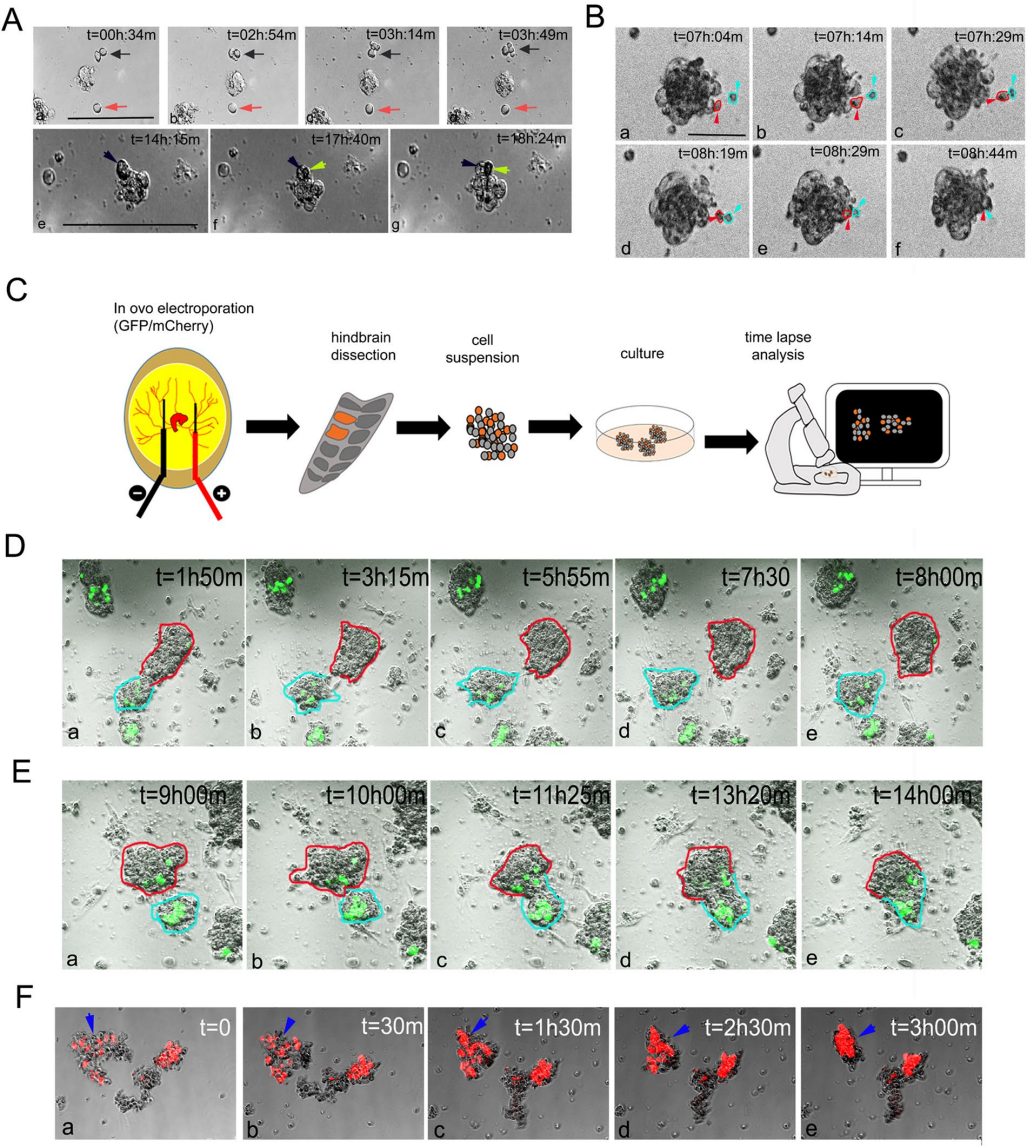
Spheres are formed via cell proliferation, cell recruitment, clustering, separation and compaction. (**A,B)** Time-lapse analysis of hindbrain cell cultures seeded in low density (100 cells/ml) and documented one day later for 18 hrs. A single dividing cell (Aa–d, black arrow), a non-dividing cell (Aa–d, red arrow), cell dividing in a newly formed aggregate (Ae–g, black and green arrows), and recruitment of a single cell to a newly formed sphere (Ba–f, blue and red arrows/circles) are shown. (**C)** Scheme of experimental design showing dissociation and culturing of cells from st.18 HH chick hindbrains following electroporation with GFP/mcherry plasmids. Cells were seeded at a density of 1.5 × 10^5^ and documented one day later for 18–24 hrs. (**D**–**F)** Time-lapse captures of electroporated hindbrain cell cultures show separation of single sphere into two distinct spheres (Da–e, red and blue circles), fusion of two aggregates into a single entity (Ea–e, red and blue circles) and compaction of sphere (Fa–e, blue arrow). Cells were electroporated with GFP (**D**,**E**) or mcherry (**F**) plasmids. In all analyses, consecutive time points of live imaging movies are indicated. Each image is a representative of three different cultures from three biological repeats. Each biological repeat included dissection of 35–40 embryonic hindbrains. Scale bar = 100 um.

The composition of the growth medium that enables the maintenance or the differentiation of NSCs *in vitro* is fundamental^6^. To determine which type of medium is adequate for hindbrain NSCs to form neurospheres, hindbrains from st.18 HH chick embryos were separated into single cell suspension (5 × 10^4^ cells/ml) and grown for 14 days in either standard tissue culture medium or embryonic stem cell (SC) medium (Fig. 1A, exp.I). Media were replenished every 3 days. During the first 2 days of incubation, small free-floating aggregates could be seen in both conditions (Fig. 1Ba,d). Yet, aggregates in the standard medium were small and few cells also adhered to the plate and begun to extend processes (Fig. 1Ba), as compared to larger floating aggregates that were observed in the SC medium (Fig. 1Bd). Following 7 and 14 days of incubation, the spheres grew in size in both conditions. However, in the standard medium the spheres adhered to the plate and developed extensive neurites or collapsed and generated monolayers with typical neuronal morphology (Fig. 2Bb,c). At variance, most spheres in the SC media remained free-floating and retained rounded and compact with almost no extension of neurites (Fig. 2Be,f). This experiment confirmed the ability of hindbrain-originating cells to form typical free-floating aggregates that tend to either adhere/collapse or to maintain as spheres, depending upon the media.

Additional support for this observation came by analyzing the ability of neurospheres to preserve their structure by incubating them for 1 month in SC medium, with or without its replenishment (Fig. 1A, exp II). Upon replacement of the medium every 3 days, the neurospheres continued to grow in size while remained rounded and free floating (Fig. 1Bh). Yet, when the neurospheres remained in their original medium, they gradually collapsed, adhered and formed a monolayer (Fig. 1Bg). This result may suggests that without refreshing the medium, the spheres are allowed to secret differentiation-promoting factors, whereas medium replenishment supports their stem/spherical structure^6,32^. Together, these findings validate the preferred conditions (i.e., SC medium replaced every 3 days) to grow and maintain hindbrain-derived spheres *in vitro*; these conditions were used for the next sets of experiments, unless described otherwise.

Cell density is a known factor to affect culture behavior^33^. We next compared the ability of hindbrain cells to form spheres or to collapse and propagate as monolayers upon cell concentration (Fig. 1A, exp III). Cell suspension was diluted into three concentrations (5 × 10^3^ cells/ml, 5 × 10^4^ cells/ml, 1.5 × 10^5^ cells/ml), and grown for 14 days without replenishing the media. In the diluted culture, single cells or very small clusters could be found after 1 day (Fig. 1Ca). Two weeks later, small adherent spheres with extended neurites or monolayers could be found (Fig. 1Cd). In the intermediate dilution, smaller and larger aggregates as well as single cells were initially evident (Fig. 1Cb). A mixture of floating spheres, adherent spheres with neurite outgrowth and monolayers were present after two weeks (Fig. 1Ce). In the condensed culture, numerous small and large aggregates were formed already after one day (Fig. 1Cc). These clusters turned with time into large floating spheres with typical rounded shape and almost no cellular extension (Fig. 1Cf), indicating that in dense conditions, replenishing the media is not necessary in order to maintain spheric. This may be a result of cells not secreting differentiation factors or not responding to such signals in a dense environment due to the rapid aggregation and compaction of cells (see also Fig. 2), that shifts the balance towards NCS propagation instead of differentiation, as opposed to cells growing in lower density. Overall, these data indicate that the ability of hindbrain primary cultures to give rise to stable neurospheres or to differentiate with time is influenced by the growth media and cell density.

### Hindbrain-derived neurospheres display dynamic cell division, aggregation and movement

Once we demonstrated the different properties of hindbrain-derived neurospheres with regard to cell density, we aimed at tracing their formation. As a hallmark of NSCs is to self-renew, a main hypothesis is that single hindbrain NSC can propagate to initiate sphere-formation^34^. We have recently corroborated this by showing the ability of hindbrain-derived primary spheres to form secondary spheres^25^. Another possible scenario for neuroshpere assembly can be via recruitment of cells with similar properties^35^. Support for this possibility comes from the observation of the rapid appearance of floating aggregates after only a few days in the culture (Fig. 1), which cannot be solely attributed to cell division. To distinguish between these two (not mutually exclusive) scenarios, we utilized time-lapse imaging. First, we determined whether cells divide in primary spheres. Hindbrain-derived cells were plated in a diluted concentration (100 cells/ml), which is necessary to test clonal capacity^10,34^. Cultures were imaged a day later for 18 hours. Free-floating cells could be documented during division (Fig. 2Aa–d, black arrow; see Supplementary Video S1). Non-dividing cells were also observed (Fig. 2Aa–d, red arrow). Proliferating cells could also be documented within a newly-formed cluster (Fig. 2Ae–g, green arrow; see Supplementary Video S2). In parallel, isolated cells were also found to join an existing sphere (Fig. 2Ba–f, blue arrow; see Supplementary Video S3). This occurred via a detachment of a cell from an existing cluster, its contacting to a remote cell, and their co-merging into the sphere (Fig. 2B, red and blue arrows).

To monitor further the sphere’s cell dynamics, we labelled cells by electroporating hindbrains of st.15 HH embryos with GFP/mCherry-expressing plasmids. Hindbrains were harvested at st.18 HH and cultured for one day prior to their tracing by live–imaging (Fig. 2C). To gain rapid formation of spheres we favored the use of the higher cell concentration (1.5 × 10^5^ cells/ml). Along with our demonstration of cell proliferation and recruitment into spheres (Fig. 2A,B and data not shown), this analysis revealed several other phenomena; cell clusters were found to detach from one aggregate and to form a new entity (Fig. 2Da–e, red and blue circles; see Supplementary Video S4). Moreover, several separated aggregates fused and established a larger sphere (Fig. 2Ea–e, red and blue circles; see Supplementary Video S5). In addition, floating aggregates were found to undergo compaction that led to the formation of condensed and rounded sphere (Fig. 2Fa–e, blue arrows; see Supplementary Video S6).

**Figure 3:**
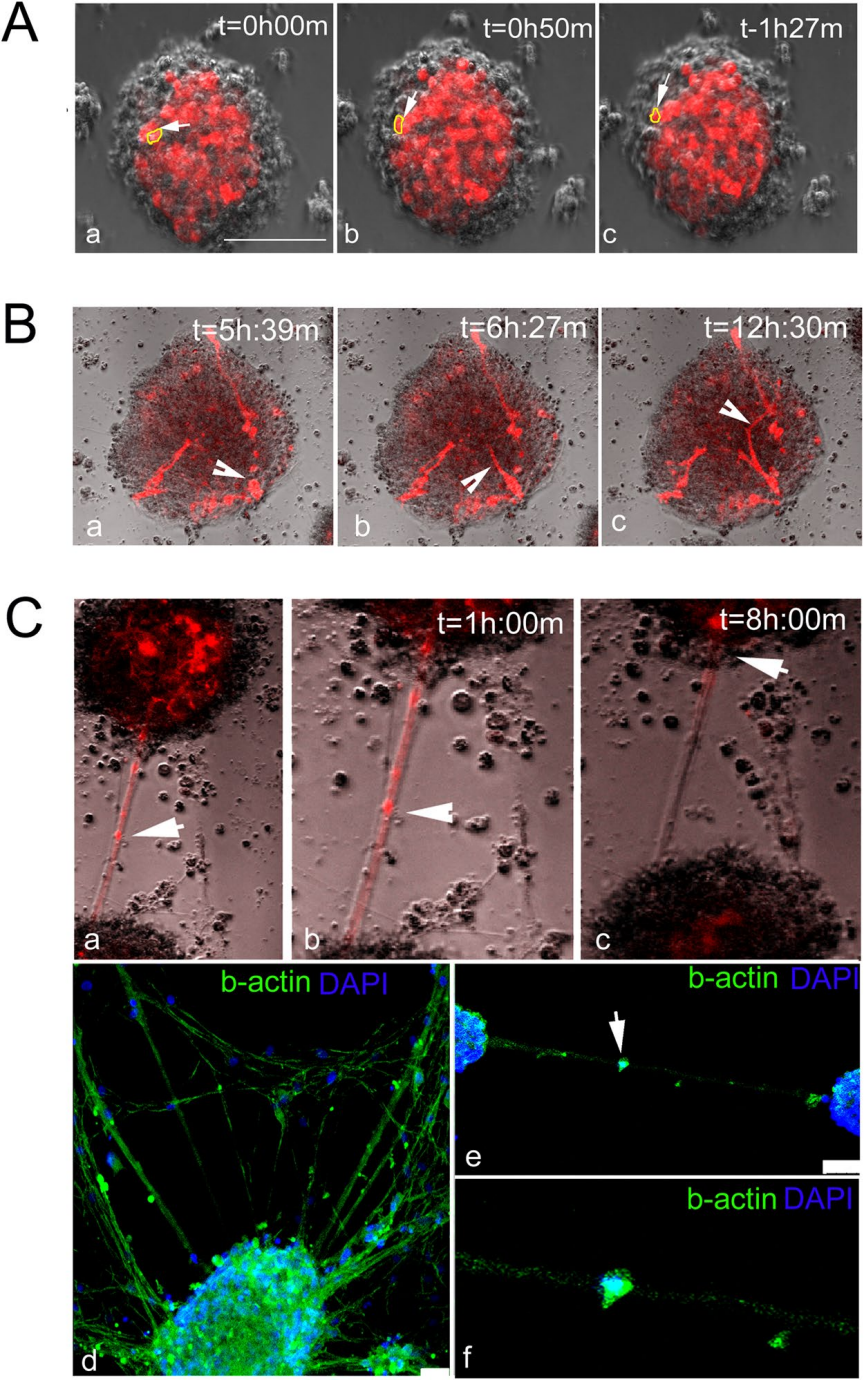
Cell movement, neurite outgrowth and network formation in hindbrain spheres. (**A**–**C)** (a–c) Time-lapse documentation of hindbrain cells that were electroporated with mCherry and cultured for four days before monitoring. (**A**) A cell migrating within a sphere (a–c, arrow and circle). (**B**) Extension of a neurite on the sphere surface (a–c, arrowhead). (**C**a–c) Connection between two spheres (a,b; low and high magnification, respectively). A cell moving along an inter-spheric process is shown (b,c, arrow). In all images, captured times are indicated. Bar = 100 um. (**C**,d–f**)** Immunofluorescence staining for b-actin marks the inter-spheric connection (green, b-actin; blue, DAPI). A cell positioned along the b-actin process is shown (e,f, arrow). Bar = 50 um. Each image is a representative of three different cultures from three biological repeats. Each biological repeat included dissection of 35–40 embryonic hindbrains.

Cell dynamics was also imaged following three more days in culture. Cells were found to constantly change their location within a given sphere (Fig. 3Aa–c, white arrow/yellow circle; see Supplementary Video S7), as well as to grow neurites that extend on its outer surface (Fig. 3Ba–c, white arrowhead; See Supplementary Video S8). Moreover, many adjacent neurospheres were found to connect via cellular extensions (Fig. 3Ca–c). These bridges express the cytoskeletal marker b-actin (Fig. 3Cd–f). Interestingly, cells seemed to migrate along these cytoskeletal cables (Fig. 3Cb,c,e, white arrows; see Supplementary Video S9). Taken together, these analyses suggest that hindbrain-derived spheres can form via both self-renewal and cell recruitment, as well as demonstrate highly dynamic cellular movements and interactions within single spheres or between neighboring ones.

### The molecular properties of hindbrain-derived neurospheres

**Figure 4:**
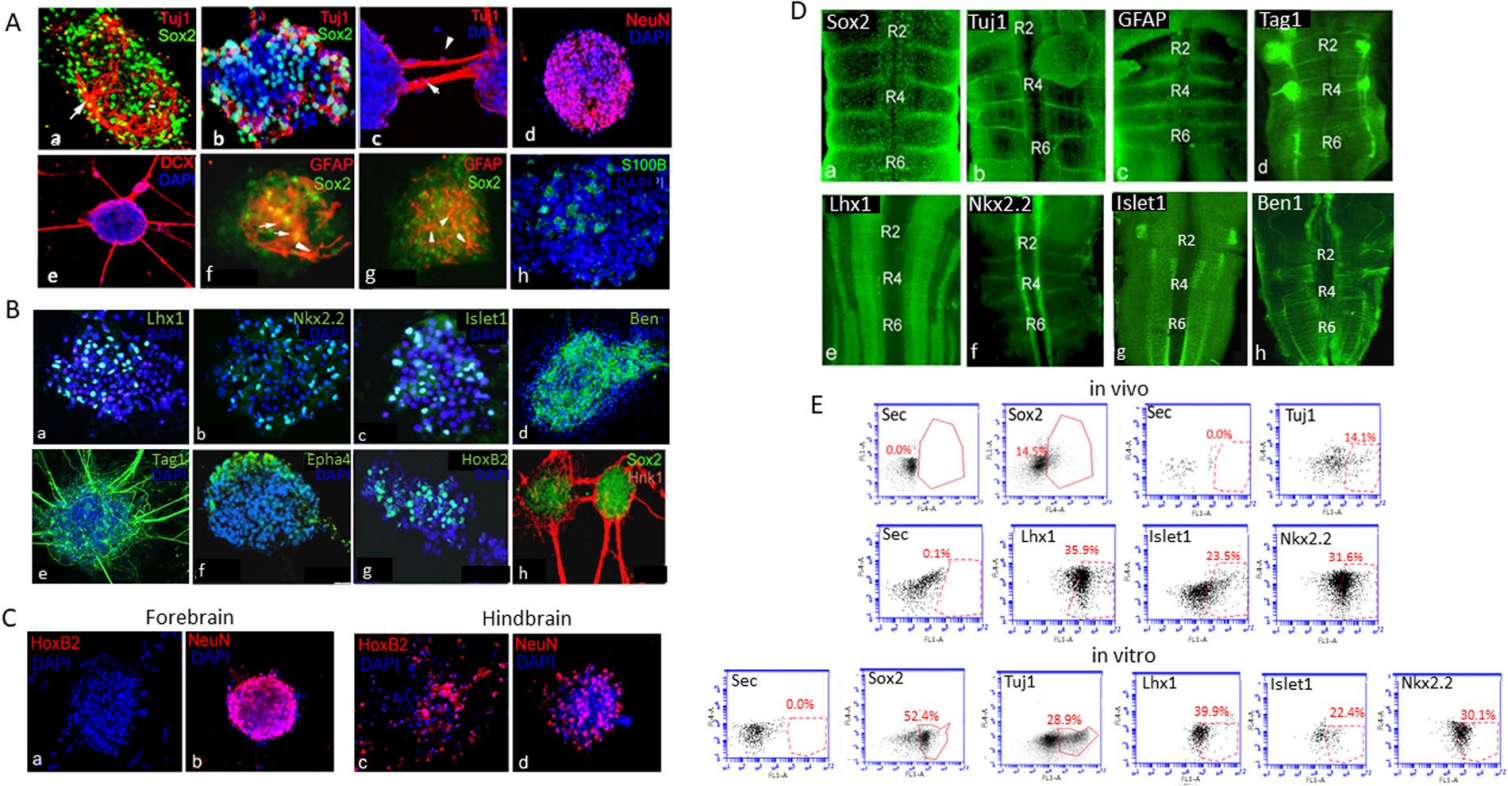
Hindbrain neurospheres are heterogeneous and multipotent and retain their regional identity. (**A**–**C**) Immunofluorescence staining of hindbrain (**A**–**C**) or forebrain (**C**)-derived cultures with different antibodies to analyze progenitor markers (Sox2, GFAP), neuronal markers (Tuj1, NeuN, DCX, Tag1, Nkx2.2, Islt1, Lhx1, Ben), glial markers (GFAP, s100B), and hindbrain markers (EphA4, HoxB2, HNK1). In all images, antibodies are indicated in their respective channels. Cell nuclei is marked by DAPI (blue). Images were taken from whole-mounted spheres except from (Ab, Ah, Ba,b,c,f,g, Ca,c) which were taken from cryo-sections. Each staining is a representative of three different cultures from three biological repeats, each included dissection of 35–40 embryonic hindbrains or forebrains. Bar = 50 um. (**D**) Flat-mount views of whole hindbrains immunostained for various progenitor/differentiation markers (green). Antibodies are indicated. Each image is a representative of 10 embryos. R = rhombomere; Bar = 50 um. (**E**) Flow cytometry analysis of cells obtained from intact hindbrains (*in vivo*) or hindbrain-derived cultures (*in vitro*). Cells were labelled with varuious antibodies, as indicated in each plot. Each plot is a representative of three whole hindbrains or three different cultures from three biological repeats, each included dissection of 35–40 embryonic hindbrains. As a negative control cells were stained only with secondary antibody (Sec). Fraction of labelled cells is encircled and precentage is indicated in each plot.

Our live tracing data demonstrated that cells within neurospheres extend neurites, divide, move, adhere, detach or remain immobile (Figs 2 and 3, see Supplementary Videos S1–S9). The molecular identity of these varied cells had to be explored. We recently showed the expression of a few progenitor and differentiation markers in hindbrain cultures^25^. Here we analyzed in greater depth the molecular properties, proliferation state and regional identity of hindbrain-derived neurospheres. Examination of the NSC marker Sox2^36,37^ revealed marked distribution of Sox2 throughout neurosphere’s cells (~50% cells/sphere), indicating the presence of stem/progenitor cells in this multicellular entity (Fig. 4Aa,b, Table 1). Different types of early or late neurogenesis markers; NeuN, Tuj1, and doublecortin (DCX)^38,39^, were also found to be expressed within neurospheres (Fig. 4Aa–e). Notably, at variance from Sox2, these markers were mostly located at the sphere’s periphery, as expected from differentiating neurons^8^. In particular, the neuron beta-tubulin marker Tuj1, which was largely absent from Sox2^+^ cells and found in ~22% cells/sphere (Fig. 4Aa–c; Table 1), and the microtubule marker DCX (Fig. 4Ae), were mostly evident in neurites extending from the sphere. These cellular extensions also formed networks that connected neighboring spheres (Fig. 4Ac), consistent with the live imaging results (Fig. 3C, see Supplementary Video S9). In addition to the expression of early and late neuronal differentiation markers, hindbrain-derived neurospheres can also give rise to glia. Glial fibrillary acidic protein (GFAP) is a well-known marker that labels both embryonic NSCs and differentiated astrocytes^40,41^. Examination of GFAP and Sox2 expression revealed that some GFAP^+^ cells were co-labelled with Sox2 (Fig. 4Af, arrow), confirming further the presence of NSCs within the sphere. Yet, GFAP was also expressed in Sox2-negative cells, which displayed short neurite extensions at the neurosphere’s outer layers (Fig. 4Af,g, arrowhead), consistent with glial-cell morphology. Testing the expression of S100b, a calcium-binding protein which labels glia-derived astrocytes^42^, revealed the existence of ~20% of cells expressing S100b^+^ in spheres (Fig. 4Ah arrows, Table 1). Together, these staining indicate a typical combination of stem/progenitor cells and differentiating neuronal and glial cells within neurospheres. The distribution of these markers in the hindbrain-derived neuropheres fits well with their expression *in-vivo*; staining of st.18HH intact hindbrains revealed their expression in the embryonic hindbrain (Fig. 4Da–c, see also^25^).

**Table 1:** Percentage of marker-expressing cells in hindbrain-derived neurospheres.

Marker	No. of total cells in a neurosphere	No. of marker expressing cells in a neurosphere	% of marker-expressing cells
Sox2 (n = 5)	236	124	51
Tuj1 (n = 5)	343	78	22
Islt1 (n = 5)	85	25	29
Nkx2.2 (n = 5)	87	23	26
S100b (n = 3)	174	37	21
EphA4 (n = 5)	129	21	17
HoxB2 (n = 5)	115	33	29
Lhx1 (n = 4)	96	29	30

Data represents proportion of cells expressing a specific marker out of total cells in one representative neurosphere. Counting was performed in 5–10 distinct spheres. Number of total cells was determined by counting DAPI-stained nuclei. Number of marker-expressing cells was determined by counting immunofluorescence-labelled cells.

As Tuj1, NeuN and DCX are general neuronal markers, we also examined markers for specific neuronal subpopulations. The homeobox protein Nkx2.2 and the basic helix-loop-helix gene Islt1 are two transcription factors which are expressed in the ventral hindbrain (Fig. 4Df,g) and essential for the development of motor neurons and ventral interneurons, respectively^43,44^. Both markers were expressed in a subset of cells (~25%) within the sphere (Fig. 4Bb,c, Table 1). Lhx1 belongs to the Lim homeodomain family of transcription factors that is expressed in dorsal hindbrain interneurons (Fig. 4De), which give rise to several sensory neuronal lineages *in vivo*^44^. Its expression was also seen in ~30% of neurospheric cells (Fig. 4Ba, Table 1). Notably, all these markers are known to be expressed in early specified neurons. Hence, we wished to analyze whether additional markers that label fully-differentiated sensory/motor neurons are also expressed in neurospheres. Ben (DM-GRASP), a surface glycoprotein of the immunoglobulin superfamily, is a known marker for motoneuronal axons^45^. Its labeled axonal projections in the hindbrain *in vivo* (Fig. 4Dh), as well as found in neurospeheres (Fig. 4Bd). Tag1 is a glycoprotein expressed commissural/sensory axons as well as in ganglia exit points^46–48^. It was found to be expressed in hindbrain axonal fibers *in vivo* (Fig. 4Dd), as well as in neurospheres (Fig. 4Be). Notably, both Ben and Tag1 labeled cells in the outer surface of neuropsheres and in extending axons. These patterns, which are similar to those found with the general axonal markers Tuj1 and DCX (Fig. 4Aa,c,e), suggest that dorsal and ventral hindbrain interneurons and motoneurons are capable to develop and mature in culture. Altogether, these data confirm that hindbrain-derived neurospheres display a stereotypic stem-to-differentiation hierarchy *in vitro* that can give rise to both neuronal and glia cell lineages. Moreover, as the expression of different types of markers in neurospehres corresponded well with the presence of these molecules *in vivo* (Fig. 4D, see also^25,44^), this finding strongly supports the retaining of original hindbrain cell properties in the *in vitro* conditions.

To compare between the percentage of cells expressing stem/neuronal markers in the culture and in intact hindbrains, st.18 hindbrains and 7 day-old cultures were analyzed via flow cytometry (Fig. 4E). The NCS factor Sox2 and the neuronal marker Tuj1 were expressed in ~15% of hindbrain cells *in vivo* (see also^25^). In neurospheres, the fraction of Sox2^+^ and Tuj1^+^ cells increased to ~50% or ~28% of cells, respectively. These results indicate for a possible selection for stem/progenitoral cells in the neurospheres, that tend to initiate their neural differentiation in the *in vitro* conditions. Yet, number of cells expressing specific neuronal markers remained largely similar in the *in vivo* and *ex vivo* samples (Nkx2.2 ~30%; Islet1 ~23%; Lhx1 ~37%), indicating that the specification of cells within hindbrain-derived neurospheres into different neural fates is maintained. Notably, data obtained from the flow cytometry analysis was comparable to our counting of labelled cells within spheres (Table 1), supporting further the typical distribution of stem/progenitorial/differentiated cells in hindbrain-derived neurospheres.

An important question is whether hindbrain-derived spheres retain their regional identity *in vitro*. HoxB2 is a transcription factor essential for the specification of several hindbrain rhombomeres^49,50^. EphA4 is a tyrosine kinase receptor that is expressed in rhombomeres 3, 5 and prevents cell mixing across rhombomeres^51–53^. Both markers were expressed within neurospheres (Fig. 4Bf,g). Counting of cells within spheres revealed that ~17% or ~29% of cells expressed HoxB2 and EphA4, respectively (Table 1). Another unique hindbrain characteristic is the complementary expression of Sox2 and the sugar epitope HNK1 (CD57) in st.18 embryos^25^; HNK1 is expressed in all rhombomeres but excluded from their boundaries whereas Sox2 is restricted to boundaries (see Supplementary Fig. S1). To test whether hindbrain-derived spheres recapitulate the same boundary/rhombomere expression patterns *in vitro*, neurospheres were stained for both markers. Clear segregation was found between Sox2^+^ cells, that were mostly located at the sphere’s core, and HNK1^+^ cells that populated the outer layer of the spheres and it’s extending neurites (Fig. 4Bh), in agreement with the *in vivo* patterns.

To further verify the hindbrain-specific identity in the culture, we compared between hindbrain and forebrain-derived neurospheres. For this experiment, both regions were isolated from the same embryos and cultured for 7 days in similar densities and growth conditions. Staining with HoxB2 demonstrated clear labeling of hindbrain spheres (Fig. 4Cc, see also Fig. 4Bh), but not forebrain spheres (Fig. 4Ca). Yet, both cultures were labelled with the general neuronal marker NeuN (Fig. 4Cb,d), as expected from neurospheres originating from various neural tube regions. Overall, these data strongly suggest that progenitor cells collected from the embryonic hindbrain maintain expression of hindbrain markers in culture and retain their original identity.

**Figure 5:**
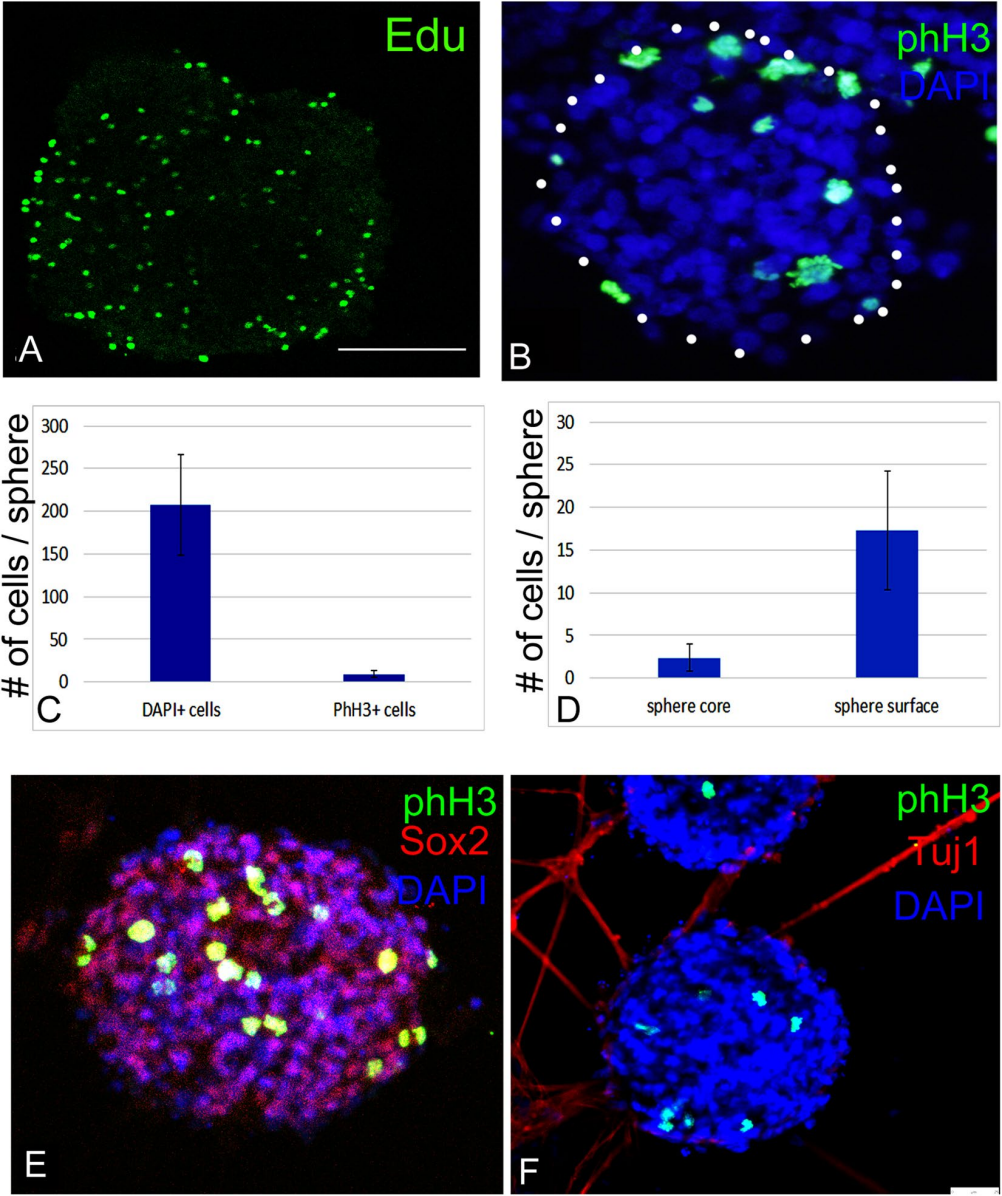
Proliferation state of hindbrain-derived neurospheres. (**A,B,E,F**) Immunofluorescence staining of hindbrain neurospheres with EdU (**A**, green), phH3 (**B**,**E**,**F**, green), Sox2 (**E**, red) and Tuj1 (**F**, red). Nuclear staining is indicated by DAPI in blue (a,c,d). Images were taken from whole-mounted spheres except in B, which was taken from a cryo-section. In all panels, antibodies are indicated in their respective channels. Each staining was repeated three times from three biological repeats. Each biological repeat included dissection of 35–40 embryonic hindbrains. Bar = 50 um. (**C**) Quantification of the number of phH3-labelled cells within a sphere (n = 16 spheres). (**D**) Quantification of the number of phH3-labelled cells within the sphere’s core versus its periphery (n = 20 spheres).

Typically, neurospheres should contain a mixture of quiescent/slow proliferating NSCs, amplifying progenitors, dividing and post-mitotic differentiating neurons^54^. To examine the proliferation state of hindbrain cultures, neurospheres were stained for phosphorylated histone 3 (phH3) and 5-ethynyl-2′-deoxyuridine (EdU), that mark cells in the M or S phase of the cell cycle, respectively^55,56^. Most of the cells were negative for both markers (Fig. 5A,B), reflecting a low mitotic index of this cell population. Notably, fewer cells were labeled with phH3 compared to Edu, as expected from cells in S or M phase of the cell cycle. Counting of cells revealed that ~5% cells/sphere expressed phH3 (Fig. 5C). This low number of dividing cells within neurospheres implies that the majority of the cells are either quiescent NSCs or post-mitotic differentiating cells. Noticeably, fewer proliferating cells (~10%) were seen within the core of the sphere compared to its outer surface (Fig. 5A,B,D). The spatial pattern of cell division correlates well with the transition from stem to differentiation state, which is displayed from the neurosphere’s core to its external domains (Fig. 4, and^5^). Further support for this conclusion came by staining for phH3 and Sox2 (Fig. 5E); the majority of Sox2^+^ cells did not express phH3, indicating for the large presence of quiescent/slow proliferating NSCs. The fewer cells that expressed both markers are likely to represent amplifying Sox2^+^ progenitors. Staining for Tuj1 and phH3 demonstrated the Tuj1 is expressed in the neurite-extending cells that did not express phH3 (Fig. 5F), as expected from post-mitotic differentiating cells. This analysis confirmed that hindbrain-derived neurospheres display an expected combination of proliferating and quiescent cells, as previously shown *in vivo*^14,15,25^.

### Segregation of different types of hindbrain cells reveals distinct stem/differentiated cell state in culture

**Figure 6:**
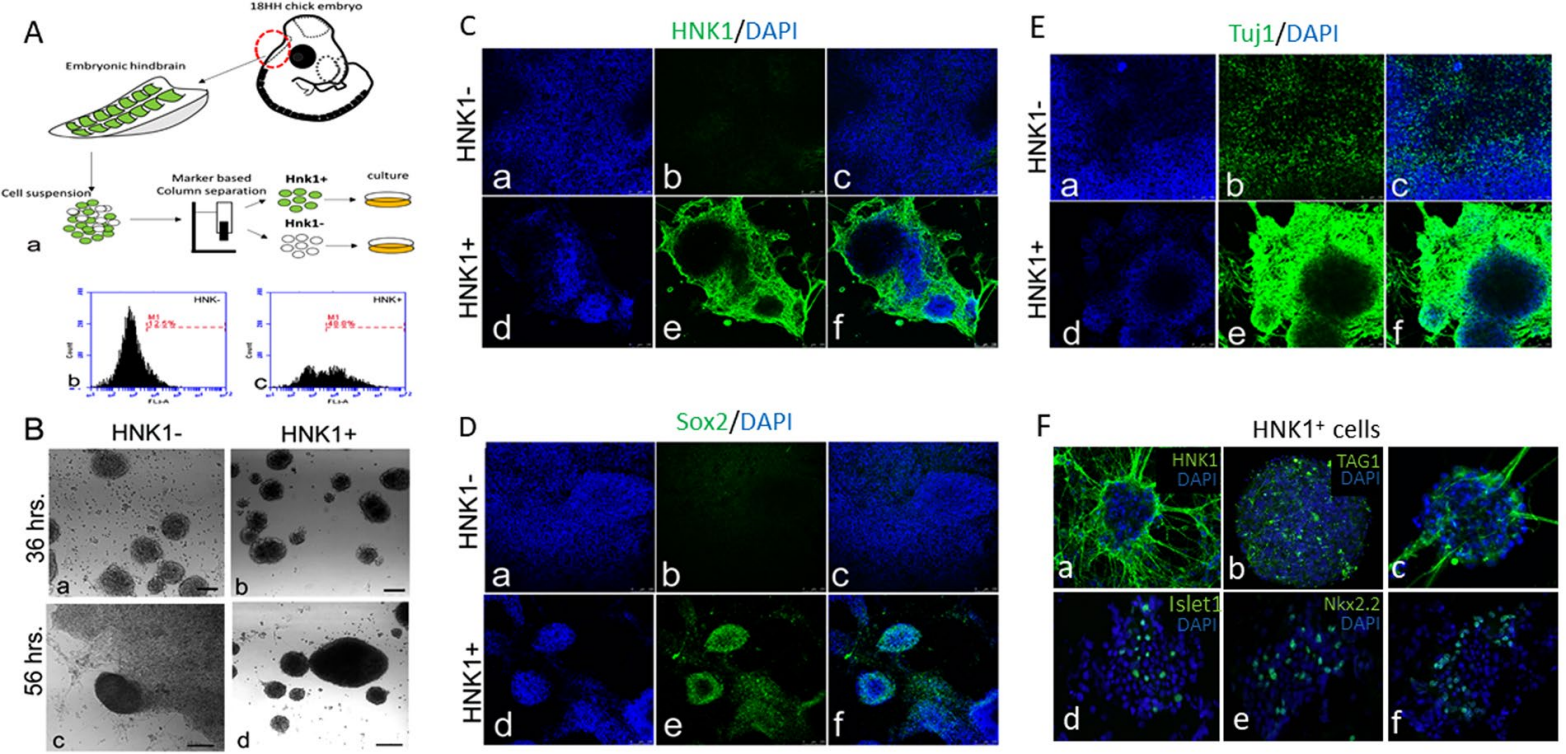
Segregation between HNK-1 positive and negative cells reveals different neurosphere properties. (**A**) (a) Schemtaic illustratuion of the magnetic-coloumn sepration assay of hindbrain cells based on expression of HNK1. (b,c) Flow cytometry analysis showing enrichment of HNK1 labelled cells in the HNK1^+^ cell fraction (b) compared to the HNK1^−^ cell fraction (a). (**B**) Bright-field views of HNK1^−^ (a,c) and HNK1^+^ (b,d) cultures shown 36 hr and 56 hr post separation. (**C**–**E**) Immunofluresence staining of HNK1^−^ (a–c) and HNK1^+^ (d–f) -derived cultures. Cells were stained for HNK1 (**C**, green), Sox2 (**D**, green) and Tuj1 (**E**, green). Nuclear staining is indicated by DAPI (blue). (**F**) Immunofluresence staining of cultures obtained from HNK1^+^ isolated cells. Cells were stained for HNK1 (a, green) or multile neuronal markers (b–f, green). Nuclear staining is indicated by DAPI (blue). Each separation experiment included dissection of ~60 hindbrains. Each experiment included three biological repeats. Bar = 50 um.

In st.18 embryos, the ventricular layer of all rhombomeres expresses HNK1. At variance, the mantle layer of rhombomeres as well as the boundaries in-between rhombomeres are devoid from this marker^25^ (see also Supplementary Fig. S1). Notably, neural crest cells, which also express HNK1, already detached from the dorsal hindbrain at. st.10–13, hence, they are not present in our analysis. Here we wanted to determine how the ventricular/non-ventricular hindbrain cell subpopulations develop in culture. Based on the ventricular expression of HNK1 we isolated live cells using an anti-HNK1 antibody on a magnetic immuno-column cell separation system^25,57^. This strategy allows to segregate HNK1^+^ from HNK1^−^ hindbrain cells (Fig. 6Aa). Flow cytometry analysis confirmed marked increase (from ~12% to ~50%) in HNK1- expressing cells in the eluted sample compared to the flow-through fraction (Fig. 6Ab,c). Culturing the two cell fractions for several days revealed distinct growth patterns; at first both cell populations developed clear floating spheres (Fig. 6Ba,b). Yet, only in the HNK1-negative fraction single rounded cells as well as adherent ones that began to form neurites, were also seen (Fig. 6Ba). A day later, the HNK1-negative fraction largely adhered to the plate and developed a typical neuronal monolayer (Fig. 6Bc). In contrast, the HNK1^+^ population continued to grow into floating spheres. Even when some spheres adhered and grew neurites, they largely maintained their typical spheroid shape (Fig. 6Bd). To further confirm the purity of the cell separation, cultures were stained for HNK1. While the monolayer deriving from the HNK1-negative fraction did not express this marker (Fig. 6Ca–c), the HNK1^+^ fraction displayed clear expression of this epitope in the spheres (Fig. 6Cd–f). Notably, HNK1 was localized to the sphere’s periphery, consistent with data shown in Fig. 4C. To characterize the stem/differentiation state of the two cell groups, cultures were stained for Sox2 and Tuj1. The HNK1^+^ fraction displayed marked expression of Sox2 within the neurosphere’s core (Fig. 6Dd–f) and Tuj1 in its outer surface (Fig. 6Ed–f), as expected from a typical neurosphere. In contrast, the monolayer deriving from HNK1-negative cells did not express Sox2 (Fig. 6Da–c) but expressed Tuj1 (Fig. 6Ea–c). Moreover, gene expression analysis was performed on the two cell fractions by real-time PCR and demonstrated that the HNK1^+^ cell fraction expressed higher levels of pro-neural genes, such as NeuroD1 and Pax6, compared to the HNK1^−^ fraction (see Supplementary Fig. S2). Together, these results, which present a useful strategy to separate different types of hindbrain cells, suggest that the progenitorial properties of the rhombomere’s ventricular layer and the differentiated state of non-ventricular other hindbrain cells are conserved in culture.

Whether neurospheres obtained from the entire hindbrain (Fig. 4) and from the HNK1^+^ cell population (Fig. 6) display similar neuronal properties? Staining of HNK1^+^ cultures revealed that the spheres, which expressed HNK1 as expected (Fig. 6Fa, see also Fig. 6Cd–f), contained cells expressing different neuronal markers; a subset of cells expressed the dorsal interneuron marker Lhx1 and the commissural/sensory axonal marker Tag1 (Fig. 6Fb,f), while other cells expressed the ventral interneuron/motoneuron markers Islet and Nkx2.2, as well as the axonal protein Ben (Fig. 6Fc–e). This analysis confirms that HNK1^+^-derived spheres can give rise to several neural cell types which are comparable to the properties found in whole-hindbrain derived neurospheres (Fig. 4). The similarities between neurospheres from these two cellular origins strongly suggest that the majority of spheres obtained from whole-hindbrain (Fig. 4A,B) are composed of cells from the ventricular/HNK1^+^ hindbrain layer (Fig. 6).

## Discussion

We recently identified a new source of hindbrain NSCs that resides in-between rhombomeres *in vivo*^25^. Here we continued to explore how well these and other types of hindbrain-derived cells can grow in culture while retaining their stem-to-differentiation hierarchy and hindbrain characteristics. For this purpose, we developed methods to isolate, expand, manipulate, sort and trace hindbrain-derived cells *in vitro*. We demonstrated the ordered formation of multipotent self-renewing neurospheres that maintain their regional identity and contain cells with different stem/quiescence/proliferation/differentiation properties. These cultures were found to reproduce major aspects of hindbrain development *in vitro*. Live imaging analysis revealed dynamic cellular movements within and between neurospheres, confirming the active state of hindbrain-derived NSCs. Combining the large body of knowledge regarding hindbrain development together with our ability to simulate the development of hindbrain progenitors in culture, opens the way for better investigating the fate and cross-talk of different CNS-cell subpopulations *in vitro*

While sources for mammalian NSCs from embryos or adults are reasonably accessible in terms of quantity and availability, these sources are still somewhat limited. Also, in many cases they are based on cell lines, mostly from rodents, and therefore may not fully represent the *in vivo* environment^29,58–61^. These facts dictate that new sources of NSCs are constantly in need. The embryonic chick model provides a highly convenient answer for these concerns. Chick embryos have been used for many decades in research since they are amniotes and therefore serve as a suitable model for early vertebrate development in comparison to mammalian embryos^62–67^. Embryos are highly accessible, relatively simple to handle, and advantageous in terms of ethical permits. Utilization of genetic tools to insert and manipulate genes of interest as well as to generate genetic-engineered lines are greatly expanding^68–74^. All these propose this model as a powerful experimental system for purifying and propagating NSCs in order to study basic neurodevelopmental questions *ex vivo*.

Current literature describes two main processes by which NSC cultures can grow. One way is via formation of a two dimension (2D) adherent monolayer, where NSCs tend to form clusters termed rosettes, followed by their differentiation into various lineages^34,75^. Alternatively, NSCs form stereotypic free-floating 3D neurospheres that are composed of stem/progenitor cells with the ability to differentiate into neurons and glia^6,8,76^. We found that hindbrain stem/progenitor cells choose to give rise to neurospheres, although no specific measures were taken to prevent their adherence. These neurospheres follow classical steps to retain self-renewal capacity together with giving rise to neurons/glia. Interestingly, environmental factors, such as type of growth medium, frequency of medium renewal, and cell density, all shift the cultures from neurospheric/stem-like structures to adherent/differentiating monolayers. These findings are in agreement with previous works where similar conditions greatly affected the culture’s fate^8,33,77,78^.

The ordered development of hindbrain cultures into spheres is somewhat surprising since the harvested hindbrains are composed of a mix of stem, progenitor, early-differentiating and differentiated cells^25^. Yet, the majority of cells tended to form 3D spheres that largely express Sox2. This is somewhat different from other systems, such as NSCs from mice embryonic SVZ, where less amount of cells give rise to neurospheres in culture^4^. This tendency suggests that the growth conditions used in our system are favorable for the stem/progenitor subpopulation, which cluster and propagate as neurospheres, as opposed to more differentiated cells. Alternatively, we cannot exclude the possibility that some early-differentiating cells are able to shift back to a more progenitorial state in the dish. Switching between stem/progenitorial and differentiation states was also gained via segregation of specific hindbrain subpopulations; rhombomeric-ventricular layer/HNK1^+^ cells tended to develop neurospherers whereas the rest of hindbrain cells generated neuronal monolayers. Notably, we recently reported that hindbrain boundaries *in vivo* contain a reservoir of quiescent and amplifying Sox2^+^ NCSs. The amplifying cells were found to move to the rhombomeres and contribute Sox2+ progenitor cells to the rhombomere’s ventricular zone^25^. Hence, it is likely that the trend of the HNK1^+^ cell fraction to form neuropsheres in culture is due to the presence of Sox2^+^ proliferating cells, which were contributed, at least partially from the boundaries. Moreover, while most of the HNK1^−^ cells fraction consists of non-ventricular/differentiating neurons, hindbrain boundary cells, which are devoid of HNK1, should also be present in this cell fraction. Yet, the fact that culturing of the HNK1-negative cell group gave rise to monolayer neurons may suggest that the majority of differentiating neurons in the sample dominated the much lower number of boundary cells, and shifted the dish into differentiation *in vitro*. Alternatively, it is also possible that the boundaries cross-talk with the adjacent rhombomeres in order to maintain their noon-differentiating properties, which was lost in cell fractions. Together, these results demonstrate further the suitability of the chick model to isolate and study the developmental program of specific NSCs and other neuronal subpopulations *in vitro*.

The majority of NSC researches *in vitro* focus on the clonal capacity and differentiation of 3D neurospheres^8,26,76–81^. Our live imaging data revealed dynamic cell movements and interactions within one sphere or in neighboring ones. Early-aged aggregates constantly fuse or depart from each other, prior to their compaction. Moreover, single cells propagate to form newly formed spheres, or are being recruited to an existing aggregate in an ordered manner. Marked cytoskeletal networks were also found to develop between spheres, which allowed inter-cell trafficking. Interestingly, several lines of evidence emphasized the significance of cytoskeletal bridges that are formed by NSCs *in vivo* to ensure their proper migration and differentiation^82–84^. Hence, it remains to be explored whether similar cell/sphere dynamics exist in other types and origins of NSCs and what can be the role of these intensive cellular behaviors *in vitro* and *in vivo*.

Finally, our findings confirm that hindbrain-derived neurospheres can give rise to several types of neuronal lineages and to glia. Such properties confirm the nature of the cultures as containing multipotent NSCs, as widely accepted in the literature^26,84,85^. Moreover, our cultures retain typical hindbrain landmarks. This result is compatible with other studies where NSCs from different neural tube domains and species preserved their original identity and gave rise to specific neuronal subgroups^28,86^. Altogether, these properties strength the feasibility of the chick embryonic system as a system to study specific NSC subpopulations.

## Methods

All embryonic work and experimental protocols were carried out in accordance with the guidelines and approval of the Authority for Biological and Biomedical Sciences and the Department of Safety and Occupational Health of the Hebrew University.

### Embryos

Fertile Loman Broiler chicken eggs (Gil-Guy Farm, Israel) were incubated in standard conditions at 38 °C for 3.5 days until reaching stage 18 Hamburger Hamilton (st.18 HH). Embryonic work was carried out along the guidelines and approval of the Hebrew University of Jerusalem, Israel.

### Primary cell cultures

Embryonic chick hindbrains and forebrains were obtained by dissecting the relevant domains of the embryonic neural tube of st.18 HH embryos. Dissected tissues were incubated in enzyme solution (TrypLE Express, Gibco, USA) for 10 minutes (min) at 37 °C. Tissue was further dissociated into single cell suspension by manual pipetting. TrypLE was neutralized by adding standard media (1:1 DMEM/F-12, (Gibco, USA), 10% fetal bovine serum (Sigma-Aldrich USA), penicillin-streptomycin (1:50; Gibco, USA) and amphotericin B (1:400; Sigma-Aldrich, USA). Cells were passed through cell strainer (100 um) to eliminate aggregates and plated in different dilutions in a 24-well Nunclon Delta Surface culture plate (Thermo Scientific, USA) with standard media (as described above) or embryonic stem cell (SC) media (DMEM/F-12 1:1, 20% KnockOut serum replacement, 2 mM GlutaMax L-alanyl-L-glutamine, 0.1 mM non-essential amino acids (all from Gibco, USA), 0.1 mM β-mercaptoethanol (Sigma-Aldrich, USA), penicillin-streptomycin (1:50) and amphotericin B (1:400)). Cells were incubated in standard conditions (37 °C/5% CO_2_). Unless stated differently, media was refreshed every 72 hours (hr).

### Cryo-sectioning

Neurospheres were collected from 7-day-old cultures and fixed in 4% paraformaldehyde (PFA, Sigma-Aldrich, USA) for 0.5 hr in room temperature (RT). Neurospheres were incubated for overnight (ON) in 30% sucrose (Sigma-Aldrich, USA) in PBS at 4 °C and embedded in Optimal Cutting Temperature compound (Sakura Finetek, USA) using a cryomold. Frozen blocks were sectioned using a CM1860 cryostat (Leica, Germany) and mounted onto slides for immunofluorescence staining.

### EdU labeling

Labeling with 5-ethynyl-2′-deoxyuridine (Edu) labeling was performed as described in Click it™ EdU Alexa Fluor™ 488 imaging kit (Invitrogen, USA). Briefly, hindbrains from st.18 HH embryos were dissected and dissociated with TrypLE Express for 10 min at 37 °C. Cells were washed with PBS solution and incubated for 2 hr with Click it™ Edu solution, as provided in the kit. Following incubation, cells were fixed in 4% PFA and permeabilized using 0.5% triton in PBS solution. Cells were washed twice with PBS and incubated for 30 min in Click it™ cocktail solution which contains Alexa Fluor® and azide. Cells were washed twice with PBS, mounted onto slides and subjected to fluorescent microscopy imaging.

### In ovo electroporation

Embryos of st.15 HH were injected with pCIG-GFP or pCIG- mCherry plasmids (1.5–3 ug/ul) onto the hindbrain lumen and electroporated using L-bent gold electrodes (1 mm diameter) in ECM 830 electroporator (BTX, Harvard Apparatus, USA). Electroporation parameters included four 45-millisecond pulses of 18–20 volts with pulse intervals of 300 milliseconds. Embryos were incubated for 24 hr prior to isolate the hindbrains and prepare single cell suspension^21–25^.

### Magnetic bead cell separation

Separation of subpopulations of hindbrain cells was conducted using MACS micro-bead cell separation kit (Miltenyi Biotec, Germany), according to the manufacturer’s protocol with minor modifications^25,57^. Briefly, hindbrain cell suspension was obtained from st.18 HH embryos and incubated with mouse anti-CD57 (HNK1) antibody (1:400, #560844; BD Biosciences, USA) for 60 min at RT. After applying washing solution (PBS/0.5% BSA), cells were incubated with anti-mouse IgG micro-bead-conjugated antibody solution for 30 min at 4 °C (1:10, #130-048-401, Miltenyi Biotec, Germany). Cells were washed and loaded on MACS magnetic columns to enable the CD57^+^ cells to attach to the column and the CD57^−^ cells to elute. Subsequently, CD57^+^ cells were removed from the magnetic field and eluted using mild force. Isolated cell populations were plated and incubated in SC media as described before.

### Flow Cytometry

Whole hindbrains dissected from st.18 embryos, or 7-days old hindbrain cultures were fixed in 4% PFA and centrifuged at 600 g for 15 min. Cells were washed in PBS/0.01% Triton X-100, centrifuged and incubated ON at 4 °C in PBS/1% BSA with primary antibodies (1:300). Following washes and centrifugation, cells were incubated for 2 hr at RT in PBS/1% BSA with Alexa-Fluor secondary antibodies (1:300), washed, and re-centrifuged for flow cytometry analysis. As a negative control, samples were exposed to secondary antibodies only. Cells were passed through an Accuri-C6 flow cytometer (BD Biosciences, USA). Data was analyzed using BD-Accuri C6 software^25^.

### Immunofluorescence staining

Cultures were fixed in 4% PFA/PBS for 15 min at RT. Next, cultures were incubated in PBS/0.01% Triton X-100 (Sigma-Aldrich, USA) for 15 min and washed with PBS for 5 min twice. Cells or cryo-sections were incubated with primary antibodies (all diluted in PBS/1% BSA) for 2 hr at RT. List of antibodies includes: Mouse-anti CD57 (1:400; #560844; BD Biosciences, USA), Rabbit-anti Sox2 (1:400; #ab5603; Millipore, USA), Mouse anti-Tuj1 (1:400, #ab14545; Abcam, UK), Mouse anti-GFAP (1:400; #IF03L; Calbiochem, USA), Rabbit anti-phH3 (1:400; #sc-8656-R; Santa Cruz Biotechnology, USA), Mouse anti-NeuN (1:400, #MAB377, millipore), Mouse anti S100b (1:200; #Ab41548, Abcam), Goat anti DCX (1:200, #SC-8060 Santa Cruz Biotechnology, USA), mouse anti-Islt1 (1:25; #40.2D6, DHSB, University of Iowa, USA), Mouse anti-Nkx2.2 (1:25; #74.5A5, DHSB, University of Iowa, USA), mouse anti-HoxB2 (1:25; #PCRP-HoxB2-1C9, DHSB, University of Iowa, USA), mouse anti-BEN (1:300; DHSB, University of Iowa, USA), mouse anti Lhx1 (1:50; #4F2, DHSB, University of Iowa, USA) and EphA4 (1:100; #SC-921; Santa Cruz Biotechnology, USA). Following several PBS washes, cells were incubated with anti-mouse/rabbit/goat Alexa-Fluor 488/594 secondary antibodies (1:500, Thermo Fisher, USA) for 2 hr at RT. Cultures were washed with PBS and incubated with DAPI nuclear staining (1:1000 Sigma-Aldrich, USA) for 10 min, washed again and incubated in PBS at 4 °C for microscopy analysis. For whole-mount staining, fixed embryos were washed and incubated in PBS with 0.1% Tween 20/5% goat serum (Biological Industries, Israel) for 2 hrs at RT for blocking, before incubation with primary antibodies for ON (same dilutions as above). Following washes, embryos were incubated for 2 hrs at RT in PBS/0.1% Tween20/5% goat serum with secondary antibodies (1:500). Following washes, hindbrains were stained with DAPI and flat-mounted on slides for microscopy analysis.

### Time-lapse analysis

Culture plates were incubated for up to 18 hr in a closed chamber at 38 °C/5% CO_2_. Cultures were magnified with Plan-Apochromat × 10 objective and captured every 5 min using inverted confocal microscope (CTR 4000) with DFC300FXR2 camera (Leica, Germany). Time-lapse movies were composed of 3–6 frames/second using Leica LasX software and processed in Adobe premiere software.

### Real time PCR

Real-time reverse-transcriptase (RT)-PCR was performed on cDNA prepared from HNK1^+^ and HNK1^−^ cell groups. mRNA was isolated immediately after cell separation in the immuno-columns using RNAqueous®-4PCR kit, according to the kit protocol (Life Technologies, USA). cDNA was prepared using High Capacity cDNA Reverse Transcription kit (Applied Biosystems, USA). PCR was performed using SYBER reagent with the following primers: Achaete-Scute Complex-Like 1 (ASCL1): fw 5′-CTTTAGCACCGACGTGTCTTAC-3′ rv 5′-GCTCTTCTGCTGTTGGACAAT A-3′; Brain Derived Neurotrophic Factor (BDNF): fw 5′-CGGGACTCTTGAAAGCCTAAC-3′ rv 5′-GCTGGATGTCCTGATCTTCATC-3′; Paired Box 6 (Pax6): fw 5′-CCGCACATGCAGACA CACATGAAT-3′ rv 5′ TCACTGCCAGGAACTTGAACTGGA-3′; NeuroD1 (ND1): fw 5′-TAACCTCGCGATGGGCCAATTACA-3′ rv 5′-ATAATTCGGCGCGGGAAAGGTAAC-3′. Results were normalized to GAPDH, as described elsewhere^32^ and analyzed using StepOne Software v2.2.2.

### Data analysis and imaging

Cells or sectioned were imaged using CTR 4000 confocal microscope and DFC300FXR2 camera (Leica, Germany). Z-stack images were generated using Leica Microsystems software. Whole-mounts were imaged using Axio Imager M1 microscope with MRM camera (Zeiss, Germany). Quantification of the expression of the different markers was determined by counting marker-expressing cells within whole neurospheres and calculating the ratio between the labeled cells and total cells (as evaluated by DAPI nuclear staining) in tested neurospheres. Analysis of phH3 expression in different regions of neurospheres was performed by counting cells at the periphery or core of neurospheres, out of the entire neurosphere cells. In all cases, counting of stained cells was conducted using the lasX Leica Microsystems software. Statistical analysis was performed using Excel software (Microsoft, USA).

## Electronic supplementary material

Supplementary legends and figures

Supplementary movie 1

Supplementary movie 2

Supplementary movie 3

Supplementary movie 4

Supplementary movie 5

Supplementary movie 6

Supplementary movie 7

Supplementary movie 8

Supplementary movie 9

## Data Availability

Materials and protocols available upon request from the corresponding author.
