# Supplementary material for: Neural stem cells deriving from chick embryonic hindbrain recapitulate hindbrain development in culture
Source: Sci Rep. 2018 Sep 17;8:13920. doi: 10.1038/s41598-018-32203-w (PMC6141497; doi:10.1038/s41598-018-32203-w)

`

SUPPLEMENTARY INFORMATION

Title: Neural stem cells deriving from chick embryonic hindbrain recapitulate hindbrain development in culture

Authors: Yuval Peretz, Ayelet Kohl, Natalia Slutsky, Marko Komlos, Stas Varshavsky and Dalit Sela-Donenfeld

`

Supplementary video S1: Time-lapse analysis of a single cell dividing in a culture obtained from hindbrain cells.

Supplementary video S2: Time-lapse analysis of hindbrain derived culture. A newly formed aggregate shows cell proliferation within the cluster.

Supplementary video S3: Time-lapse analysis of cell recruitment by a newly formed sphere in a hindbrain-derived cluster.

Supplementary video S4: Time-lapse analysis of GFP- labelled hindbrain-derived culture. A separation of one sphere into two distinct aggregates is shown.

Supplementary video S5: Time-lapse analysis of GFP- labelled hindbrain-derived culture culture. A fusion of two cell aggregates into one large hindbrain-derived sphere is documented.

Supplementary video S6: Time-lapse analysis of mcherry-labelled hindbrain cell culture. Compaction of cells within existing neurospheres is shown.

Supplementary video S7: Time-lapse analysis of mcherry-labelled hindbrain cell culture. Cell movement is shown within a compacted neurospher .

Supplementary video S8: Time-lapse analysis of mcherry-labelled hindbrain cell culture. Neurite is extending on the surface of the sphere.

Supplementary video S9: Time lapse analysis of mcherry-labelled hindbrain cell culture. A cell is seen migrating on an a cable formed between two neighboring neurospheres.

Supplementary figure S1. Segregated expression of HNK1 and Sox2 in the hindbrain.

A. Flat-mount views of st.18 HH hindbrains co-immunostained for Sox2 (b, red), HNK-1 (c, green) and DAPI (a, blue). Merged image is shown in (d). (c-n) Sequential confocal Z-stack

`

views (10 to 25 μm) of similarly stained hindbrain Merged images are shown in f,j,n. n= 10 embryos

B. A model for the localization of HNK-1 in the rhombomere's ventricular zone. HNK-1 is absent from the rhombomere mantle layer as well from $Sox2^{+}$ hindbrain boundaries.

Supplemetary figure S2. Distinct expression of early and late neuronal genes in $HNK1^{+}$ and $HNK1^{-}$ cell groups.

Real Time RT-PCR was performed on cDNA prepared from $HNK1^{+}$ and $HNK1^{-}$ cell groups. Gene expression levels were normalized to GAPDH. Expression of *NeuroD1* and *Pax6*, which label early-born neurons, is elevated in the $HNK1^{+}$ cell group compared to the $HNK1^{-}$ group. Expression of *ASCl1*, which is known to promote early and late stages of neural differentiation as well as neurite outgrowth, is lower in the $HNK1^{+}$ cell group compared to the $HNK1^{-}$ group. The expression of the neurotrophic factor BDNF, which promotes the survival of different populations of developing neurons, is similar in both groups. Standard deviation was calculated using these biological repeats.

Supplementary figure 1

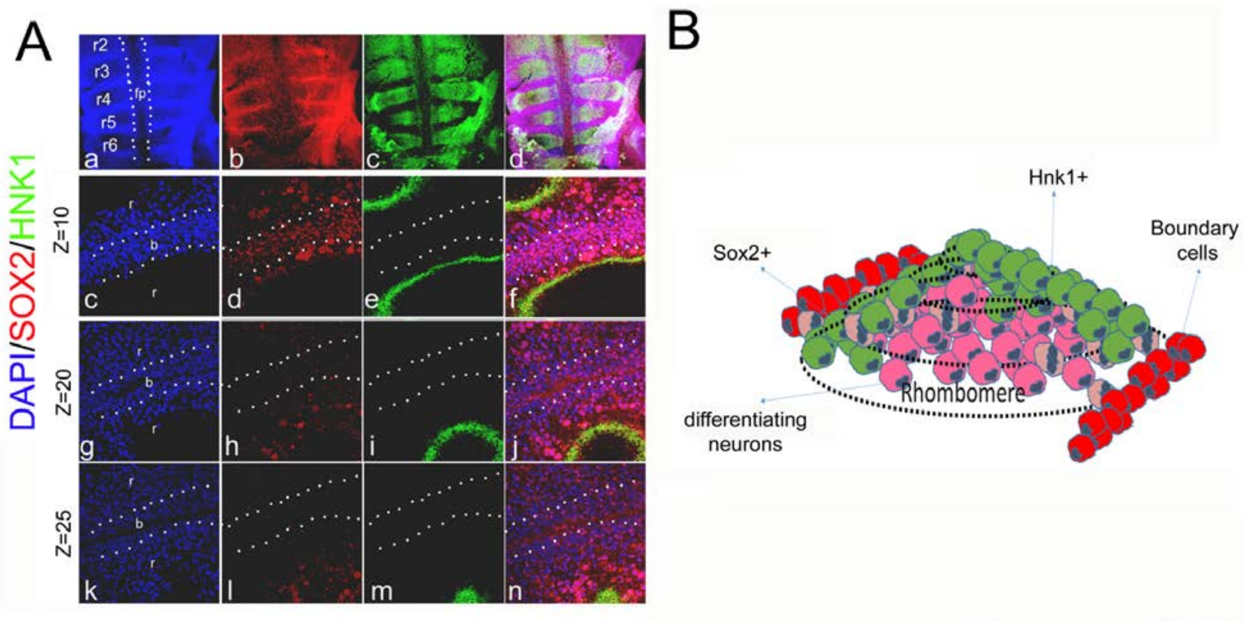

Supplementary Figure 2

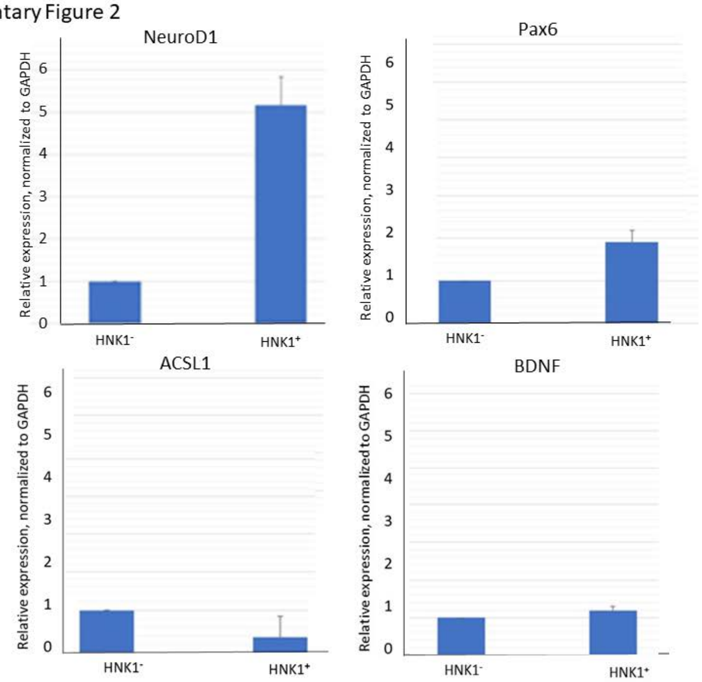

Supplement: Supplementary file 1 — Supplementary legends and figures [file 41598_2018_32203_MOESM1_ESM.pdf]
